# Clinical characteristics and epidemiological analysis of 23 cases of tick-borne rickettsiosis in Xinjiang Uygur Autonomous Region

**DOI:** 10.3389/fcimb.2026.1865543

**Published:** 2026-07-08

**Authors:** Minghui Yun, Ziqi Wang, Xiaoshuang Han, Xiangwei Wu, Chao Wei, Kaiting Zhang, Yang zhou, Xiaobo Lu, Yuanzhi Wang, Ligu Mi, Songsong Xie

**Affiliations:** 1 NHC Key Laboratory of Prevention and Treatment of Central Asia High Incidence Diseases, the First Affiliated Hospital of Shihezi University, Shihezi, China; 2 Key Laboratory for Prevention and Control of Emerging Infectious Diseases and Public Health Security of the XPCC, School of Medicine, Shihezi University, Shihezi, China; 3 Infectious Disease and Liver Disease Center, The First Affiliated Hospital of Xinjiang Medical University, Urumqi, Xinjiang, China; 4 Key Laboratory of High Incidence Disease Research in Xinjiang (Xinjiang Medical University), Ministry of Education, Urumqi, Xinjiang, China; 5 State Key Laboratory for Diagnosis and Treatment of Severe Zoonotic Infectious Diseases, Tongji Hospital, Tongji Medical College, Huazhong University of Science and Technology, Wuhan, China

**Keywords:** clinical symptoms, doxycycline treatment, laboratory tests, misdiagnosis, molecular detection, spotted fever group rickettsia

## Abstract

**Background:**

Previously, at least ten spotted fever group *Rickettsia* (SFGR) species were detected in wildlife and hard ticks in Xinjiang Uygur Autonomous Region (XUAR, northwestern China), whereas human infection remains scarcely studied.

**Methods:**

During April 2017 - May 2025, the blood samples of 70 tick-bitten patients in XUAR were collected. Their blood DNAs were individually extracted, and rickettsia-specific gene fragments were further detected by nested polymerase chain reaction (nPCR). Meanwhile, combined with sequencing of 16S rRNA V3/V4 region in blood pools and serological examination, clinical symptoms, laboratory test results and antibiotic treatment were further analyzed.

**Results:**

Out of 70 tick-bitten cases, 23 patients were confirmed to be infected with SFGR, including *Rickettsia raoultii* (n*=*15) and *R. sibirica* (n*=*8). The main clinical findings were as follows. Firstly, fever, rash, and headache were common clinical manifestations in spotted fever cases. Secondly, 7 indicators were statistically significant between pre- and post-treatment in hospitalized spotted fever cases when detecting laboratory tests. Thirdly, misdiagnosis or delayed administration of doxycycline was associated with progression to severe disease.

**Conclusion:**

In this study of 70 tick-bitten patients who visited the First Affiliated Hospital of Shihezi University from April 2017 to May 2025, the SFGR infection rate was 32.9% (23/70). Early diagnosis and timely treatment are crucial in spotted fever cases. Early administration of doxycycline is effective against SFGR infection, and delayed treatment increases the risk of severe outcomes. The methods of nucleic acid diagnosis, such as nPCR and next-generation sequencing (NGS), should be implemented especially in acute tick-bitten patients.

## Introduction

Rickettsiosis is a zoonotic disease caused by obligate intracellular *rickettsia*, typically transmitted by ticks, fleas, lice or mites (Mohamad Tahir et al., 2025). SFGR are transmitted to humans primarily through the bite of infected ticks, which inoculate the bacteria into the host dermis during blood feeding (Strobl et al., 2022). There is a great variety of clinical presentations of rickettsioses, and some pathogenic species, which cause debilitating diseases, are listed as bioterrorism agents. Members of the genus *Rickettsia* are divided into four clades, including spotted fever group (SFG), typhus group (TG), ancestral group (AG) and transitional group (TRG) (Mohamad Tahir et al., 2025). In China, at least 23 rickettisia species have been confirmed in human beings, livestock, wildlife, ticks and fleas and mites (Gui et al., 2022).

Xinjiang Uygur Autonomous Region (XUAR, northwestern China), covering 166.47 million km^2^, is known for its diversity of tick-borne diseases and is a high-incidence area for spotted fever in China (Guccione et al., 2023). Since 1984, the Chinese first SFGR (*Rickettsia jinghensis*, later identified as *Rickettsia sibirica*) have been isolated in Jinghe County, northern region of XUAR (Fan et al., 1987), at least ten SFGR species have been found in XUAR (Wang et al., 2025). However, whether all these species detected in ticks and reservoir animals can infect humans remains unclear. To date, only sporadic case reports have documented *Rickettsia aeschlimannii*, *Rickettsia conorii* subspecies *caspia*, and *Rickettsia raoultii* infections in individual tick-bitten patients in XUAR (Battisti et al., 2020; Seo et al., 2020; Cohen et al., 2021), and a systematic investigation of SFGR human infections in this region is still lacking.

Notably, Xinjiang shares borders with Kazakhstan, Mongolia, Russia, and other Central Asian regions, which are also potential endemic areas for tick-borne rickettsiosis. Previous studies have reported human infections caused by R. sibirica mongolitimonae in Asia (Santibáñez et al., 2025), and R. raoultii has been detected in Dermacentor ticks in Mongolia and Central Asia (Balážová et al., 2022; Barbiero et al., 2023). These findings suggest that the epidemiology of SFGR in XUAR may be linked to surrounding regions, and cross-border surveillance is warranted.

## Methods

### Patient

The blood samples [approximately 2 mL of blood collected into ethylene diamine tetraacetic acid dipotassium salt (EDTA-K2) containing tubes and plain tubes containing a clot accelerator] of 70 tick-bitten patients, who visited the First Affiliated Hospital of Shihezi University from April 2017 to May 2025, were collected. Before the conduct of this study, clinical information such as the clinical symptoms (**Table 1**), general information (**Table 2**), laboratory test results (**Table 3**), treatment plans and prognosis of the patients was systematically collected. This study was approved by the Ethics Committee of the First Affiliated Hospital of Shihezi University (Approval number: KJ2024-314-01), and all patients signed the written informed consent form to participate in this study.

**Table 1:** Demographic and clinical characteristics of 23 patients with tick-borne spotted fever rickettsiosis in Xinjiang, China.

ID	Age	Sex	Pathogen	Fever	Rash	Headache	Neuro	Severity
1	31	Male	*R. sibirica*	+	+	−	−	Mild
2	19	Female	*R. raoultii*	+	−	−	−	Mild
3	26	Female	*R. raoultii*	−	+	+	−	Mild
4	32	Female	*R. raoultii*	+	+	−	−	Mild
5	33	Female	*R. raoultii*	−	−	+	−	Mild
6	28	Female	*R. sibirica*	−	−	+	−	Mild
7	42	Female	*R. raoultii*	+	+	+	−	Mild
8	29	Male	*R. sibirica*	−	+	−	−	Mild
9	19	Female	*R. raoultii*	+	−	+	−	Mild
10	54	Female	*R. sibirica*	+	+	−	−	Mild
11	61	Male	*R. raoultii*	+	−	−	−	Mild
12	34	Female	*R. sibirica*	−	−	+	−	Mild
13	58	Female	*R. sibirica*	+	+	−	−	Mild
14	43	Male	*R. raoultii*	+	+	−	−	Mild
15	63	Female	*R. raoultii*	−	+	−	−	Mild
16	28	Female	*R. sibirica*	+	−	+	−	Mild
17	63	Male	*R. raoultii*	+	+	+	+	Severe
18	36	Male	*R. raoultii*	+	+	+	+	Severe
19†	42	Female	*R. raoultii*	+	+	−	−	Mild
20	22	Female	*R. raoultii*	−	−	+	−	Mild
21	35	Male	*R. sibirica*	−	−	+	−	Mild
22	36	Male	*R. raoultii*	+	+	+	+	Severe
23	43	Male	*R. raoultii*	+	−	−	−	Mild

Neuro, Neurological symptoms (confusion, hypersomnia, lethargy).

†Patient #19 additionally presented with retroauricular lymphadenopathy and facial edema.

Among the 23 patients, 15 were infected with *R. raoultii* and 8 with *R. sibirica*. Severe cases (#17, #18, #22) had tick bite sites on the head. Other recorded symptoms included fatigue (n=6), nausea (n=4), myalgia (n=3), cough (n=3), vomiting (n=3), abdominal pain/diarrhea (n=1), and chills (n=2).

**Table 2:** Time intervals from tick bite to symptom onset and medical consultation in 23 patients with spotted fever rickettsiosis.

ID	Pathogen	Incubation (d)	Onset to visit (d)	Severity	Admission
1	*R. sibirica*	5	1	Mild	Outpatient
2	*R. raoultii*	3	13	Mild	Outpatient
3	*R. raoultii*	11	11	Mild	Outpatient
4	*R. raoultii*	4	1	Mild	Outpatient
5	*R. raoultii*	1	1	Mild	Outpatient
6	*R. sibirica*	1	1	Mild	Outpatient
7	*R. raoultii*	6	1	Mild	Outpatient
8	*R. sibirica*	4	1	Mild	Outpatient
9	*R. raoultii*	3	3	Mild	Outpatient
10	*R. sibirica*	7	6	Mild	Inpatient
11	*R. raoultii*	4	1	Mild	Inpatient
12	*R. sibirica*	1	1	Mild	Outpatient
13	*R. sibirica*	15	10	Mild	Inpatient
14	*R. raoultii*	4	1	Mild	Outpatient
15	*R. raoultii*	5	3	Mild	Outpatient
16	*R. sibirica*	3	4	Mild	Inpatient
17	*R. raoultii*	7	3	Severe	Inpatient
18	*R. raoultii*	10	1	Severe	Inpatient
19†	*R. raoultii*	12	8	Mild	Inpatient
20	*R. raoultii*	1	1	Mild	Outpatient
21	*R. sibirica*	2	5	Mild	Outpatient
22	*R. raoultii*	10	1	Severe	Inpatient
23	*R. raoultii*	12	1	Mild	Inpatient

Incubation = time from tick bite to symptom onset.

†Patient #19 additionally presented with retroauricular lymphadenopathy and facial edema.

Median incubation period was 4 days (range 1-15). Median time from symptom onset to medical consultation was 1 day (range 1-13). Fourteen patients were treated as outpatients, nine were hospitalized.

**Table 3:** The abnormal laboratory test results of 23 cases with rickettsiosis.

Laboratory test results	Normal value	Number of cases	Prevalence rate
C-reactive protein	< 10 mg/L	11	47.82%
Erythrocyte sedimentation rate	0–15 mm/h	5	21.74%
Percentage of neutrophils	40-75%	11	47.82%
Eosinophil	(0.02-0.52)×10^9/L	10	43.47%
White blood cell	(3.5-9.5)×10^9/L	8	34.78%
Percentage of lymphocytes	20-50%	11	47.82%
Platelet	(125-350)×10^9/L	3	13.04%
Albumin	35–50 g/L	10	43.47%
Total bilirubin	<23μmol/L	2	8.70%
Lactate dehydrogenase	120–246 U/L	7	30.43%
Creatine kinase	50–310 U/L	5	21.73%
Alanine aminotransferase	Female (7-10)U/L Male (9-50)U/L	9	39.13%
Aspartate aminotransferase	Female (13-35)U/L Male (15-40)U/L	10	43.47%

### Definitions

The patients with rickettsiosis were divided into severe group and non-severe group. Severe cases were defined as patients presenting with central nervous system (CNS) involvement, including disturbance of consciousness, confusion, hypersomnia, delirium, or ptosis, based on previously published criteria for severe rickettsiosis (Li and Ma, 2023). All other cases were classified as non-severe (mild to moderate).

### DNA extraction

The ethylene diamine tetraacetic acid dipotassium salt (EDTA- K2) blood specimens of 70 tick-bitten patients were individually extracted at admission by using the Blood Genomic DNA Kit (TransGen, Beijing, China). The DNA quantity was assessed on a NanoDrop 2000 spectrophotometer (Thermo Fisher Scientific, USA). Samples with DNA concentration of at least 30 ng/μL could be used to detect pathogens.

### Serological examination

For preliminary rapid diagnosis, 55 tick-bitten patients with available serum samples (15 serum samples were unavailable due to logistical reasons) were tested using a commercial human SFGR IgG ELISA Kit (double-antigen sandwich method; Shanghai, China). Due to the limitation of single serum samples, only IgG testing was performed. Optical density (OD) was measured at 450 nm using a microplate reader. According to the manufacturer’s instructions, the cutoff value was calculated as the mean OD of the negative controls plus 0.15. Samples with an OD value ≥ the cutoff were considered positive for SFGR IgG antibodies.

### Molecular detection of common tick-borne pathogens and phylogenetic analyses

Multiple pathogens were screened by nested PCR (nPCR), including *Rickettsia* spp., *Coxiella* spp., *Borrelia burgdorferi sensu stricto, Ehrlichia* spp.*, Anaplasma* spp., *Babesia* spp., Tacheng tick virus 1, Tacheng and tick virus 2 (Wills et al., 2018; Liu et al., 2020; Dong et al., 2021; Dunaj et al., 2021; Grochowska et al., 2021; Wang et al., 2025). *Rickettsia* were detected by targeting four genetic markers, including encoding the outer membrane protein A (*omp*A), outer membrane protein B (*omp*B), cell surface antigen 1 (*sca*1) and 17-kDa antigen (Song et al., 2018).Primer sequences and cycling conditions are provided in **Supplementary Table 1**. The PCR products were purified using the TIANgel Midi Purification Kit (TIANGEN, Beijing, China) and sequenced. The obtained sequences were edited and compared to GenBank data using BLASTN program (http://www.ncbi.nlm.nih.gov/BLAST/). Maximum Likelihood (ML) Tree was further constructed for *Rickettsia* with MEGA 7.0 software (Kumar et al., 2016). Bootstrap values were obtained with 1000 replicates.

### Laboratory test

In order to compare the laboratory indicators of patients with different symptoms, and to determine whether there are statistically significant differences in these indicators pre- and post-treatment for hospitalized patients, hematological indicators and blood biochemical indicators was further performed in 70 patients. Briefly, blood samples in plain tubes containing a clot accelerator for all cases were placed into a refrigerated incubator at 4°C and transported to the laboratory within 2 h or less. The coagulated blood samples were centrifuged at 3000g for 10 min. Twenty-two hematologic analytes, such as C-reactive protein, platelet and percentage of lymphocytes were measured with the Mindray Bc-7500 Fully automatic Blood Cell analyzer. Twenty-three biochemical analytes, such as aspartate aminotransferase (AST), alanine aminotransferase (ALT) and albumin, were measured with the HITACHI Labospect 008 AS Biochemical Analyzer. Hematological and biochemical analytes measured are listed in **Supplementary Table 2**. The obtained data were analyzed using IBM SPSS Statistics version 23.0 (IBM Corporation) and GraphPad Prism 8.5.

### Statistical analysis

The Wilcoxon signed-rank test was used to compare pre- and post-treatment laboratory parameters in the 9 hospitalized patients. A two-tailed *P* value < 0.05 was considered statistically significant.

### 16S rRNA fragment sequencing for bacteria in tick-bitten patients carrying microbiome

To assess bacterial abundance and diversity in patient blood, 30 tick-bitten patients were selected, including 20 Rickettsia-positive and 10 Rickettsia-negative patients with clinical symptoms (headache, myalgia, fever ranging from 37 °C to 37.5 °C). Prior to sequencing, each 2–10 patient’s blood samples were constructed into a group according to infection status and symptoms. A total of 7 groups were finally constructed in this study (details in **Supplementary Table 3**). As detailed in **Supplementary Table 3**, samples were grouped according to two factors: nPCR-confirmed Rickettsia infection status (*R. raoultii*, *R. sibirica*, mixed, or negative) and the presence or absence of clinical symptoms at blood collection. Incubation period and time from symptom onset were not used as grouping criteria, as the sample size was too small for meaningful stratification by these variables. Library preparation and paired-end sequencing (2 × 250 bp) of the V3/V4 hypervariable region of the 16S rRNA gene were performed on the Illumina NovaSeq platform by Beijing Novogene Company (Beijing, China).

## Result

### Serological examination

Among the serum samples of 55 patients, 12 patients showed positive by serological test. The positive rate was 21.82% (12/55) (details in **Supplementary Table 4**). Notably, several patients with negative or missing serological results were subsequently confirmed by nPCR, consistent with the known delay in seroconversion during the acute phase of infection.

### Common tick-borne pathogens infection

**Figure 1:**
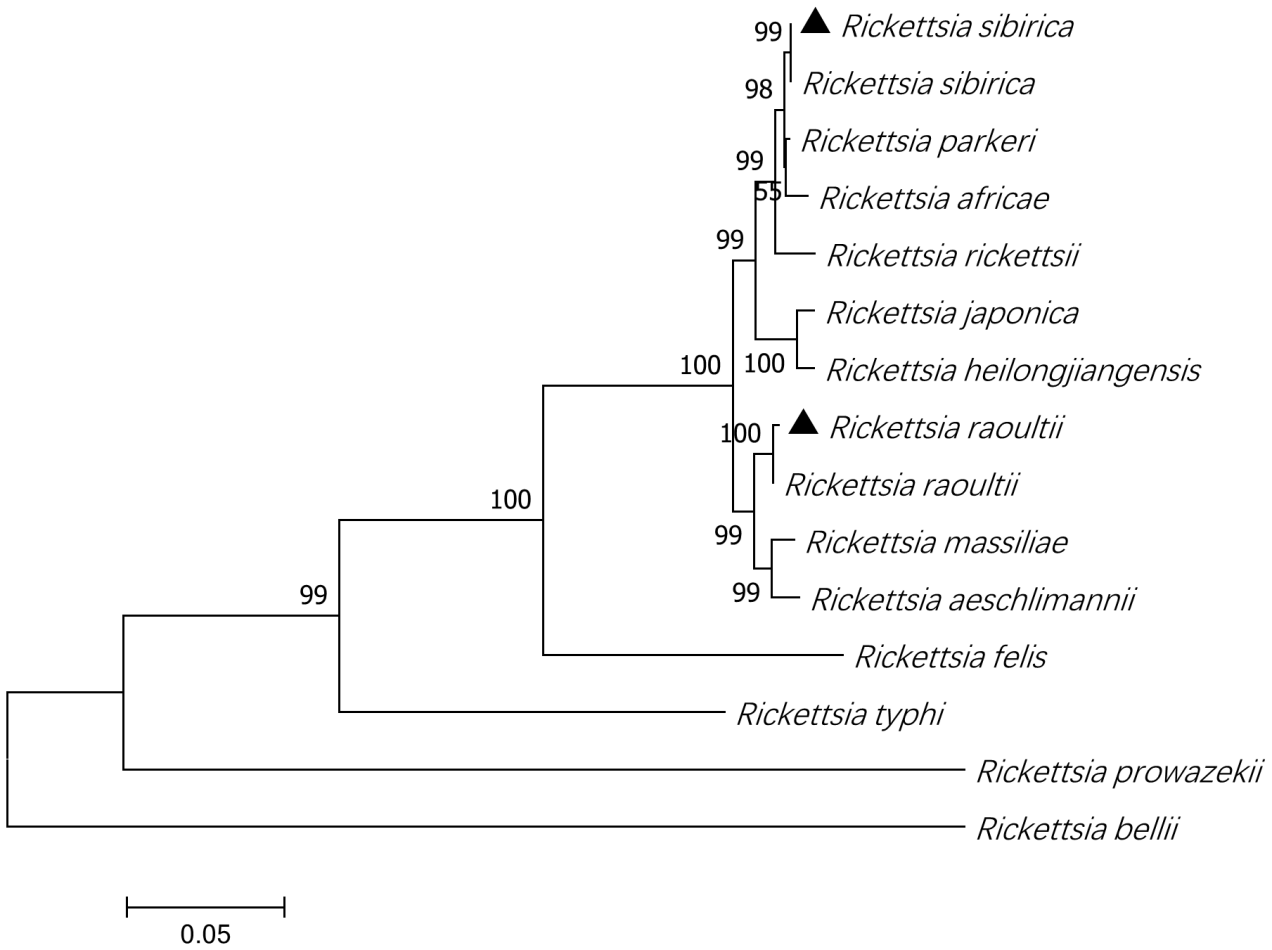
Phylogenetic analysis of rickettsia based on the tandem sequence of 17-kDa-*omp*A-*omp*B-*sca*1 genes. This phylogenetic tree was constructed based on the tandem sequences of four genes, namely 17-kDa, *omp*A, *omp*B, and *sca*1. The Maximum Likelihood (ML) method was used for the evolutionary analysis, and 1000 bootstrap replicates were performed using the MEGA 7.0 software. All sequences were first verified by comparison with the reference sequences in the GenBank database through NCBI BLAST (http://blast.ncbi.nlm.nih.gov/Blast.cgi). The *Rickettsia* sequences obtained in this study are indicated by black triangles (▲) in the figure, and *Rickettsia prowazekii* was used as the outgroup to determine the evolutionary relationship.

Among the 70 blood samples collected from tick-bitten patients who visited the First Affiliated Hospital of Shihezi University from April 2017 to May 2025, common tick-borne pathogens were negative except for Rickettsia spp. The overall PCR-positive rate for SFGR was 32.9% (23/70), including *R. raoultii* (n=15) and *R. sibirica* (n=8). After BLAST comparison, 15 sequences showed 100% identity with *R. raoultii* reference strains (GenBank accession nos.: PQ677907 for ompA, CP170620 for ompB, MF002545 for sca1, and MH071809 for 17-kDa), while the remaining 8 sequences were 100% identical to *R. sibirica* reference strains (OR117534 for ompA, ON191702 for ompB, MG811691 for sca1, and MW321546 for 17-kDa). The rickettsial sequences in this study were deposited in the GenBank database (*omp*A: PV158452 and PV258453; *omp*B: PV158454 and PV158455; *sca*1: PV158456 and PV158457; and 17-kDa: PV158450 and PV158451)(**Figure 1**).

### Clinical characteristics and epidemiologic

**Figure 2:**
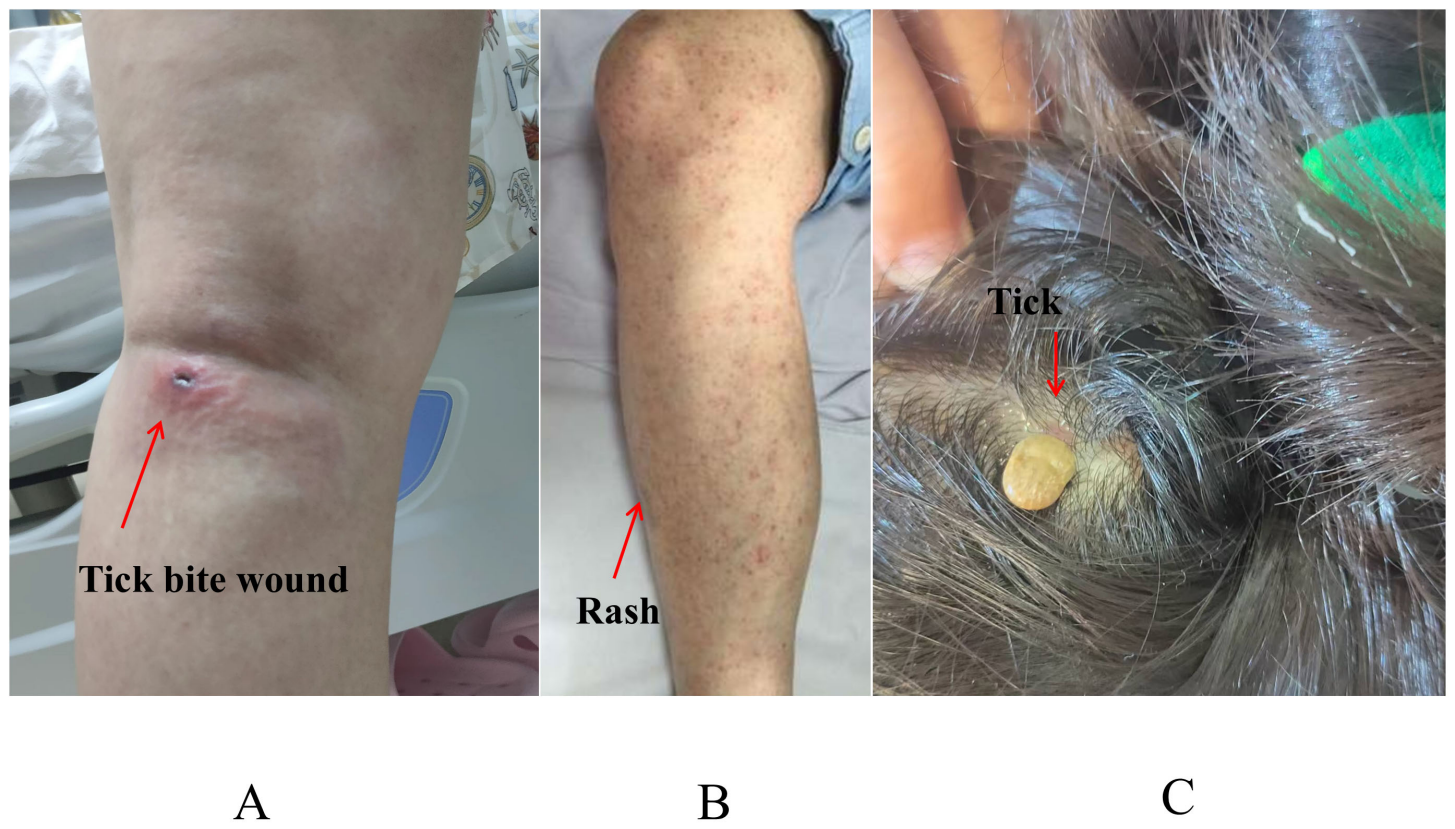
Related photos of patients bitten by ticks. **(A)** The wound formed at the popliteal fossa of the left lower leg of Case 15 due to a tick bite; **(B)** The skin rash on the right lower limb of Case 16; **(C)** The photo of the body surface of the patient bitten by a tick in Case 2.

Among the common clinical symptoms, fever (the range was from 38.2°C to 40.5°C), rash (**Figure 2B**), and headache were the most common manifestations (**Table 1**). Among the 23 PCR-confirmed SFGR cases, 3 patients (13.04%, 3/23) presented with neurological symptoms (including confusion and hypersomnia) and were classified as severe cases based on the presence of central nervous system involvement, which is one of the criteria for severe rickettsiosis according to our predefined definition (see Methods: Definitions). All 23 PCR-positive patients in this study were symptomatic, presenting with fever, rash, headache, or other clinical manifestations as shown in **Table 1**. PCR positivity in this context reflects active infection, as all enrolled patients had recent tick bites and compatible clinical symptoms. The remaining patients were classified as mild to moderate cases. In addition, patient #19 (infection of *R. raoultii*) presented with retroauricular lymphadenopathy and facial edema (**Table 1**).

Among the 23 positive patients, 20 (86.96%) were mild to moderate and 3 (13.04%) were severe. The median incubation period was 4 days (range 1-15), and the median time from symptom onset to medical consultation was 1 day (range 1-13) (**Table 2**). All 23 patients had a history of outdoor activities within one month before symptom onset. Representative images of a tick bite wound and a patient’s body surface are shown in **Figures 2A, C**, respectively.

The 23 positive cases in this study showed obvious month characteristics, which occurred from April to September. Among them, May was peak, June was followed, accounting for 34.78% (8/23) and 21.74% (5/23) of rickettsiosis cases, respectively.

In terms of treatment methods, 14 patients (60.87%, 14/23) chose outpatient or emergency treatment, and 9 patients (39.13%, 9/23) received hospitalized treatment. The clinical manifestations of the 23 positive patients in this study were highly heterogeneous (**Table 1**).

### Laboratory tests

In this study, the laboratory test results of the blood of 23 positive patients were analyzed, mainly including two aspects: hematological indicators and blood biochemical indicators, showed 13 abnormal laboratory indicators (**Table 3**).

**Figure 3:**
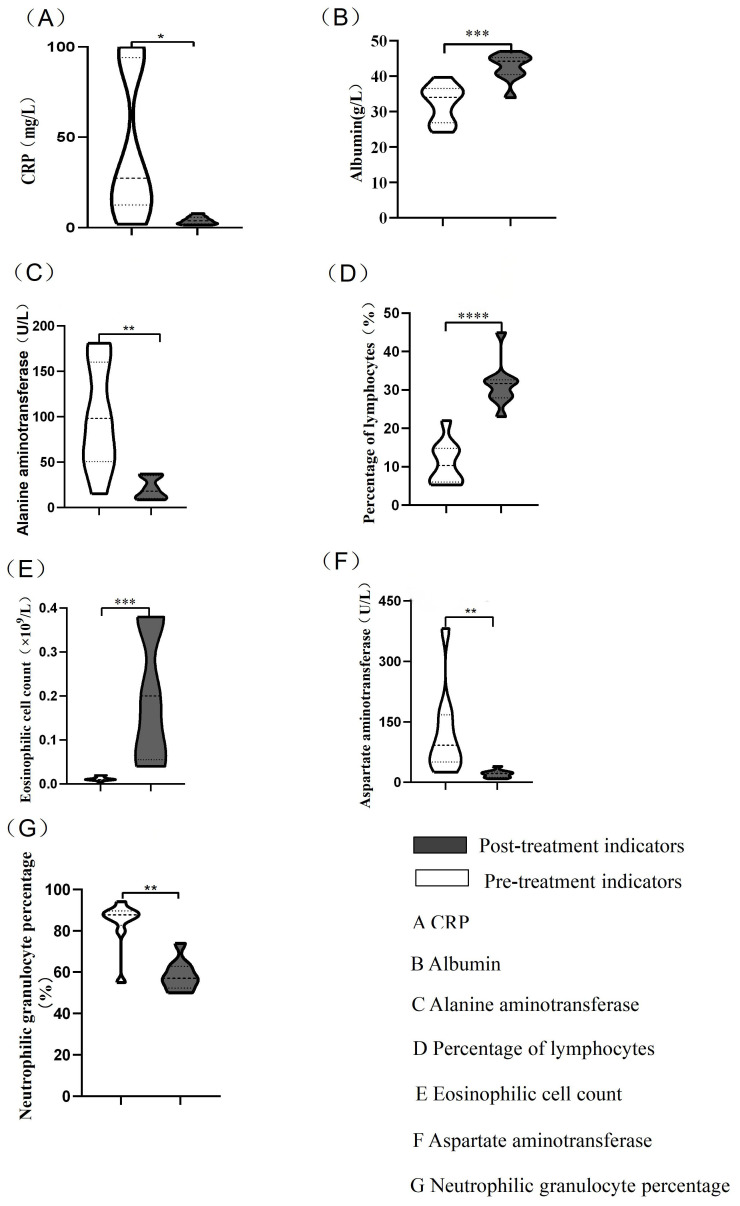
Comparison of 7 laboratory indicators showing significant differences between pre- and post-treatment in 9 hospitalized patients. Pre-treatment, post-treatment. * *P* < 0.05, ** *P* < 0.01, *** *P* < 0.001 (Wilcoxon signed-rank test). Violin plots showing the levels of **(A)** C-reactive protein (CRP), **(B)** albumin, **(C)** alanine aminotransferase (ALT), **(D)** percentage of lymphocytes, **(E)** eosinophilic cell count, **(F)** aspartate aminotransferase (AST), and **(G)** neutrophilic granulocyte percentage in patients before (white) and after (dark gray) treatment.

This study collected the laboratory test results of 9 hospitalized patients [3 severe cases (#17, #18, #22) and 6 non-severe cases] pre- and post-treatment (**Supplementary Table 5**). The results showed that all indicators returned to normal after treatment in all hospitalized patients. The Wilcoxon signed-rank test was used to compare pre- and post-treatment laboratory indicators in the 9 hospitalized patients (**Supplementary Table 5**). Among the 13 abnormal laboratory indicators used in this study, 7 indicators were statistically significant (Percentage of lymphocyte, *p* = 0.0001; Eosinophil count, *p* = 0.001; Albumin, *p* = 0.001; Alanine aminotransferase, *P* = 0.006; Percentage of neutrophil, *p* = 0.008; Aspartate aminotransferase, *P* = 0.011; C-reactive protein, *p* = 0.018). Among them, percentage of lymphocyte was the indicator that best reflected the difference pre- and post-treatment (**Figure 3**).

### Microbiome profile

A total of 7 groups (RX1-RX7) were finally constructed in this study for 16S rRNA fragment sequencing for tick-bitten patients carrying microbiome (details in **Supplementary Table 3**).

**Figure 4:**
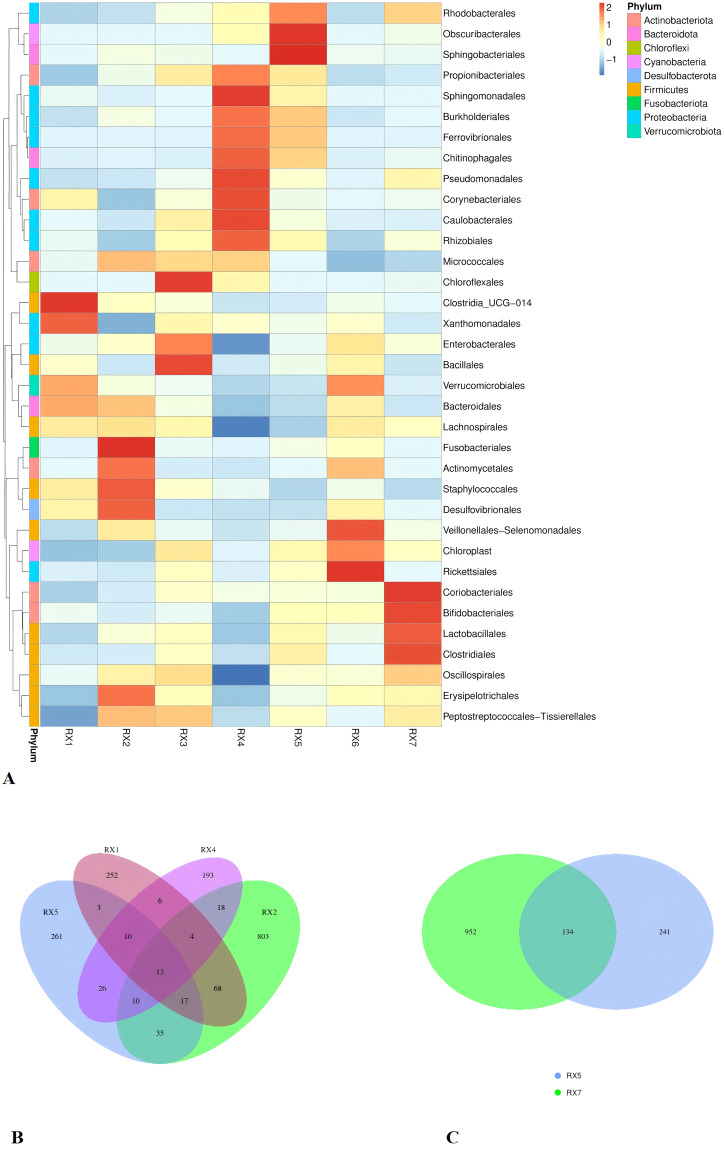
The result of 16S rRNA fragment sequencing for Bacteria in tick-bitten patients carrying microbiome. **(A)** showed the heatmap of RX1-RX7; **(B)** showed the Venn plots of RX1, RX2, RX4 and RX5; **(C)** showed the Venn plots of RX5 and RX7.

The 16S rRNA gene sequencing revealed that the relative abundance of Rickettsiales varied substantially among the seven groups (**Figure 4**). **Figure 4A** showed that Rickettsiales has the highest relative abundance in RX6 (red); The relative abundance of Rickettsiales in RX3 and RX5 is moderate (yellow). The relative abundance of Rickettsiales is relatively low in RX1, RX2, RX4 and RX7 (blue). Among them, RX1, RX2, RX4, and RX5 represented the groups infected with *rickettsia* and showing symptoms. **Figure 4B** showed the Venn plots of RX1, RX2, RX4 and RX5. **Figure 4C** showed the Venn plots of RX5 and RX7.

### Treatment and outcome

A total of 23 positive patients were included in this study, of which 14 outpatient or emergency patients were treated with oral doxycycline at a dose of 0.1g twice daily for 7 days. Telephone follow-up showed that none of the outpatient or emergency patients had any clinical manifestations after 14 days. The 9 hospitalized patients were treated with a doxycycline regimen at a dose of 0.1g twice daily for 7–10 days until fever and clinical manifestations were completely resolved.

## Discussion

XUAR lies in the region of temperate continental arid climate, where ticks are the most dominant vector. More than 19 species of *Rickettsia* have been identified in this region (Liu et al., 2021). Among them, the cases of *Rickettsia aeschlimannii*, *R. sibirica*, *R. raoultii* and *R. slovaca* infections were previously reported (Li et al., 2020; Hu et al., 2024). Although approximately 10 SFGR species have been detected in ticks in XUAR, only *R. raoultii* and *R. sibirica* were identified in human patients in this study. This discrepancy may reflect differences in host tropism or pathogenic potential among SFGR species. Other species (e.g., *R. aeschlimannii*, *R. slovaca*) may rarely infect humans, or cause subclinical or self-limiting infections that are not captured in routine clinical practice. Regarding the tick species involved in SFGR transmission in XUAR, previous molecular surveys have detected *R. raoultii* and *R. sibirica* in Dermacentor ticks collected from this region (Wang et al., 2025). We acknowledge that tick specimens were not collected from patients for species identification in the present study, which is a limitation of this clinical investigation.

The peak incidence from May to June coincides with the active period of Dermacentor ticks in XUAR, highlighting the importance of targeted prevention during these months.

Common symptoms of *R. raoultii* infection include lymphadenopathy, fever, eschar, and rash (Li et al., 2018). In this study, 15 cases with *R. raoultii* infection were found. Among them, case #19 additionally presented with facial edema. Previously, *Rickettsia* could trigger inflammation and increases vascular permeability, leading to plasma extravasation and tissue edema (Silverman and Bond, 1984; Sporn et al., 1997). In addition, cases of neurological symptoms caused by *R. raoultii* are rare (Li et al., 2018; Dong et al., 2019). Here, three severe cases (#17, #18 and #22) caused by *R. raoultii* infection, exhibited obviously neurological symptoms. Among them, cases #17 and #18, which were listed as severe rickettsiosis, had tick bite sites on the head, suggesting that patients bitten on the head are more prone to neurological symptoms. *Rickettsia* directly invades the central nervous system (CNS) through the dense nerve endings in the head, bypassing the blood-brain barrier and causing neuronal damage (Dahm et al., 2016). From a virulence perspective, rickettsiae utilize actin polymerization for intracellular motility and direct cell-to-cell spread, which may facilitate penetration of the blood-brain barrier. Additionally, rickettsial lipopolysaccharide (LPS) triggers the release of pro-inflammatory cytokines (e.g., TNF-α, IL-6), increasing vascular endothelial permeability and contributing to cerebral edema and elevated intracranial pressure. Furthermore, these three severe rickettsiosis cases exhibited elevated cerebrospinal fluid (CSF) pressure, presenting a pressure of 250 mmH_2_O, 200 mmH_2_O, and 320 mmH_2_O (reference range: 80–180 mmH_2_O), with delirium, hypersomnia, apathy and right-sided ptosis. *Rickettsia* infection increases vascular permeability, triggering vascular-induced cerebral edema and leading to increased intracranial pressure. Persistent elevation of CSF may lead to brain herniation, which causes 30%-50% mortality rate (Luo et al., 2020).

In the present study, direct comparison of our clinical data (**Table 1**) revealed several differences between the two groups. Although both infections commonly presented with fever, rash, and headache, lymphadenopathy and facial edema were exclusively noted in the *R. raoultii* group (case #19). Moreover, all three severe cases with neurological symptoms (confusion, hypersomnia) were infected with *R. raoultii* (cases #17, #18, #22), suggesting that this species may have stronger pathogenicity and neurotropic potential. In contrast, all *R. sibirica* cases presented with mild illness, and no severe or neurological involvement was observed.

The symptoms of *R. sibirica* infection typically include high fever, myalgia, headache, rash and lymphadenopathy (Santibáñez et al., 2025). In this study, the clinical features of *R. sibirica* infection were as follows. Fever was observed in 4 cases (#8, #10, #13 and #16), rash in 4 cases (#1, #8, #10 and #16), headache in 2 cases (#12 and #21) and an asymptomatic early-stage case.

Rickettsial infection induces characteristic alterations in hematological laboratory parameters. In this study, seven abnormal parameters were statistically found in nine hospitalized patients … including percentage of lymphocytes, eosinophil count, albumin, alanine aminotransferase (ALT), aspartate aminotransferase (AST), percentage of neutrophils and C-reactive protein (CRP) (**Figure 3**). These changes reflect the acute inflammatory response and mild hepatocellular injury commonly seen in rickettsial infections, and their reversal after doxycycline treatment supports the effectiveness of timely antibiotic therapy. Although these biochemical alterations are non-specific, they may serve as auxiliary indicators especially combined with tick-bitten history. However, other tick-borne diseases, such as Lyme disease, anaplasmosis, and babesiosis, may also cause similar abnormalities in these parameters, which limits their diagnostic specificity.

Rickettsial infections are frequently misdiagnosed, because the clinical symptoms are nonspecific and difficult to distinguish from other febrile illnesses (Galanakis and Bitsori, 2019). Previously, Rocky Mountain spotted fever, due to misdiagnosis, led to 10%-50% mortality rate during 2009-2023 (Álvarez-Hernández et al., 2024). In this study, three of 23 rickettsial DNA-positive patients were initially misdiagnosed, leading to clinical deterioration. Patient #17, initially misdiagnosed as papular urticaria due to fatigue and rash. Antihistamine therapy was administered till syndrome deterioration and fever (≥38°C). Patient #18 presented with high fever and dizziness. The local hospital treated him only with antipyretic therapy. In this study, the mean effectively diagnostic delay among the three severe cases was 7 days (The time from admission to doxycycline treatment). The mean time from symptom onset to doxycycline administration in these three severe cases was 10 days (range: 7–13 days), based on data from **Table 2**.

The common serological method for SFGR diagnosis in clinical practice is specific IgM/IgG antibody detection, although this approach exhibits limitations in both sensitivity and diagnostic accuracy. Specific IgM antibody and IgG antibody levels respectively require 7 days and 10 days post-infection to reach detectable levels (Colavita et al., 2023). In this study, the SFGR IgG ELISA demonstrated a positive rate of 21.82%, markedly lower than molecular detection (32.9%) (details in **Supplementary Table 4**). This likely reflects the delay in seroconversion during the acute phase of infection, when antibody titers may not yet have reached detectable levels. Meanwhile, some patients without obvious symptoms tested negative by nPCR but positive by SFGR IgG ELISA, likely reflecting prior exposure to Rickettsia rather than acute infection. The median time from symptom onset to medical consultation in our cohort was only 1 day (range: 1–13 days; **Table 2**). This early sampling window further explains the low seropositivity rate, as most patients were tested before detectable antibody levels had developed. However, this comparison has limitations, as only single serum samples were available and IgM was not tested. Paired acute-convalescent sera would be needed for a more accurate assessment of serological performance.

Previous study indicated that rickettsial infection is initiated ≥ 6 h after tick bites, resulting bacteremia. On days 5 - 9, *Rickettsia* rapidly proliferates in vascular endothelial cells, and its number increases sharply. Within 9–12 days, *Rickettsia* proliferates in cells sequentially, and reach peak on day 12 (Meng and Duan, 2011). Therefore, timely diagnosis and effective treatment are important for rickettsiosis cases. In this study, bacterial genome analysis by 16S rRNA fragment sequencing were introduced in blood detection. The *Rickettsia* abundance in the RX6 group (originated from patients #4, #21 and #3) was the highest (**Figure 4A**). Their tick-bitten history respectively experienced 5 days, 11 days, and 22 days but no treatment with doxycycline. In the RX6 group, patient 3 had mild symptoms and a long incubation period (11 days) despite high Rickettsia abundance by 16S rRNA sequencing (**Figure 4A**). This is because 16S rRNA detects bacterial DNA (including non-viable organisms) rather than live bacterial load. Patient 3 may have been sampled at a later infection stage when inflammation had subsided, or had stronger host immune response. Thus, higher 16S rRNA abundance does not necessarily mean more severe disease. Therefore, nucleic acid diagnostic methods such as nPCR and NGS are particularly valuable in acute tick-bitten patients for early confirmation of SFGR infection.

In summary, the diagnostic and treatment workflow for suspected SFGR infection in our study included: clinical suspicion based on tick bite and symptoms, molecular confirmation by nPCR, and immediate doxycycline therapy (0.1g twice daily for 7–10 days).

With respect to neighboring regions of Xinjiang, *R. sibirica* is endemic in Russia (Siberia), the classic area for Siberian tick typhus, and its subspecies *R. sibirica mongolitimonae* has caused human infections in Asia (Santibáñez et al., 2025). *R. raoultii* has been detected in *Dermacentor* ticks in Mongolia and Central Asian countries, and is a confirmed pathogen of tick-borne lymphadenopathy (TIBOLA/SENLAT) (Balážová et al., 2022; Barbiero et al., 2023). Although direct human case reports from Kazakhstan and other neighboring countries are limited, the shared tick species and similar ecosystems suggest the potential presence of these pathogens, warranting future cross-border surveillance.

Regarding treatment, doxycycline (0.1g twice daily for 7–10 days) is the first-line therapy for SFGR infection. Early administration within 3 days of symptom onset is associated with rapid defervescence and favorable outcomes. For patients with doxycycline allergy, chloramphenicol or macrolides may be considered as alternatives.

For prevention, individuals engaging in outdoor activities in endemic areas (April to September, the peak tick activity season in XUAR) should take personal protective measures, including wearing long sleeves, using insect repellents, and conducting thorough tick checks after outdoor exposure. Public health education on early recognition of tick bites and SFGR symptoms is also important.

This study has several limitations. First, the sample size of confirmed SFGR cases (n=23) is relatively small, which may limit the generalizability of the clinical findings and the statistical power of the laboratory parameter comparisons. Second, serological testing was based on single acute-phase serum samples rather than paired sera, precluding definitive assessment of seroconversion. Third, Rickettsia isolation was not attempted, and therefore antimicrobial susceptibility testing could not be performed. Additionally, the molecular diagnostic methods recommended in this study (nPCR and NGS) may not be readily available in primary healthcare settings due to equipment and cost constraints. Despite these limitations, this study provides valuable clinical and epidemiological data on SFGR infection from an understudied region and highlights the importance of molecular diagnosis in acute tick-bitten patients.

## Conclusion

In this study of 70 tick-bitten patients who visited the First Affiliated Hospital of Shihezi University from April 2017 to May 2025, 23 patients (32.9%) were confirmed to have SFGR infection by nPCR, with *R. raoultii* and *R. sibirica* identified as the predominant causative agents. The clinical presentation was characterized by fever, rash, and headache, with neurological involvement observed in severe cases. Laboratory parameters, particularly lymphocyte percentage, showed significant changes between pre- and post-treatment and may serve as auxiliary diagnostic indicators. Serological testing demonstrated limited sensitivity in acute-phase diagnosis, underscoring the need for molecular methods such as nPCR and NGS for early and accurate detection of tick-borne rickettsiosis. Early diagnosis and timely administration of doxycycline remain critical for favorable outcomes.

## Data Availability

The datasets presented in this study can be found in online repositories. The names of the repository/repositories and accession number(s) can be found in the article/**Supplementary Material**.

## Supplementary material

Supplementary Table 1 Nested PCR primers and cycling conditions for detection of *Rickettsia* spp.

Supplementary Table 2 The hematologic analytes and biochemical analytes in this study. (**A)** Twenty-two hematologic analytes; (**B)** Twenty-three biochemical analytes.

Supplementary Table 3 Sample pooling strategy for 16S rRNA gene sequencing.

Supplementary Table 4 Serological examination and nPCR results of 70 patients.

Supplementary Table 5 The Laboratory Test Results of 9 hospitalized patients pre- and post-treatment.
