# Supplementary material for: Clinical characteristics and epidemiological analysis of 23 cases of tick-borne rickettsiosis in Xinjiang Uygur Autonomous Region
Source: Front Cell Infect Microbiol. 2026 Jul 8;16:1865543. doi: 10.3389/fcimb.2026.1865543 (PMC13388217; doi:10.3389/fcimb.2026.1865543)
Supplement: Supplementary Table 1 — Nested PCR primers and cycling conditions for detection of Rickettsia spp. [file Table1.docx]

**Supplementary Table S1.** Nested PCR primers and cycling conditions for detection of *Rickettsia spp*.

| Target gene | Primer | Sequence (5' → 3') | Amplicon (bp) | Cycling conditions |
| --- | --- | --- | --- | --- |
| ****17-kDa**** | 17kDa-out-F | GCTTTACAAAATTCTAAAAACCATATA | 550 | 95°C 5min; 33× (95°C 30s, 58°C 60s, 72°C 60s); 72°C 8min |
|  | 17kDa-out-R | TGTCTATCAATTCACAACTTGCCGTT |  |  |
|  | 17kDa-in-F | GCTCTTGCAACTTCTATGTT | 434 | 95°C 5min; 33× (95°C 30s, 61°C 30s, 72°C 30s); 72°C 8min |
|  | 17kDa-in-R | CATTGTTCGTCAGGTTGGCG |  |  |
| ****ompA**** | ompA-out-F | ATGGCGAATATTTCTCCAAAA | 443 | 95°C 5min; 35× (95°C 30s, 50°C 30s, 72°C 30s); 72°C 8min |
|  | ompA-out-R | AGTGCAGCATTCGCTCCCCCT |  |  |
|  | ompA-in-F | CTTAAAGCCGCTTTATTCACCACCTC | 434 | 95°C 5min; 35× (95°C 30s, 59°C 30s, 72°C 30s); 72°C 8min |
|  | ompA-in-R | CCTGTATAATTATCGGCAGGAGC |  |  |
| ****ompB**** | ompB-out-F | ACAGCTACCATAGTAGCCAG | 1063 | 95°C 5min; 35× (95°C 45s, 56°C 45s, 72°C 2min30s); 72°C 5min |
|  | ompB-out-R | TGCAGTATAGTTACCACCG |  |  |
|  | ompB-in-F | TGCTGCGGCTTCTACATT | 812 | 95°C 5min; 35× (95°C 45s, 56°C 45s, 72°C 1min10s); 72°C 5min |
|  | ompB-in-R | ACCGCCAGCGTTCCCTAT |  |  |
| ****sca1**** | SCA1-out-F | GGTGATGAAGAAGAGTCTC | 656 | 95°C 5min; 35× (95°C 30s, 56°C 30s, 72°C 30s); 72°C 5min |
|  | SCA1-out-R | CTCTTTAAAATTATGTTCTAC |  |  |
|  | SCA1-in-F | GAGGTTTGTGGATGCGTGGT | 657 | 95°C 5min; 35× (95°C 30s, 58°C 30s, 72°C 30s); 72°C 5min |
|  | SCA1-in-R | ACTGTGACTTTAGTACCGACA |  |  |

**Note:** All reactions included an initial denaturation step and a final extension step as indicated. "out" = outer primers (first-round PCR); "in" = inner primers (second-round nested PCR).
