# Supplementary material for: Clinical characteristics and epidemiological analysis of 23 cases of tick-borne rickettsiosis in Xinjiang Uygur Autonomous Region
Source: Front Cell Infect Microbiol. 2026 Jul 8;16:1865543. doi: 10.3389/fcimb.2026.1865543 (PMC13388217; doi:10.3389/fcimb.2026.1865543)
Supplement: Supplementary Table 2 — The hematologic analytes and biochemical analytes in this study. (A) Twenty-two hematologic analytes; (B) Twenty-three biochemical analytes. [file Table2.docx]

**Supplementary Table S2.** The hematologic analytes and biochemical analytes in this study.

| **Table S2A.** Twenty-two hematologic analytes | | | |
| --- | --- | --- | --- |
| Number | Check project | reference range | unit |
| 1 | white blood cell count | 3.5-9.5 | 10^9/L |
| 2 | hematocrystallin | 130-175 | g/L |
| 3 | • platelet count PLT | 125-350 | 10^9/L |
| 4 | • erythrocyte count | 4.3-5.8 | 10^12/L |
| 5 | neutrophilic granulocyte percentage | 40-75 | % |
| 6 | Percentage of lymphocytes | 20.0-50.0 | % |
| 7 | Percentage of monocytes | 3.0-10.0 | % |
| 8 | Percentage of eosinophils | 0.4-8.0 | % |
| 9 | Percentage of basophils | 0-1.0 | % |
| 10 | neutrophil count | 1.8-6.3 | 10^9/L |
| 11 | lymphocyte count | 1.1-3.2 | 10^9/L |
| 12 | Monocyte count | 0.1-0.6 | 10^9/L |
| 13 | Eosinophilic cell count | 0.02-0.52 | 10^9/L |
| 14 | Basophils count | 0-0.06 | 10^9/L |
| 15 | hematokrit | 0.40-0.50 | L/L |
| 16 | mean corpuscular volume | 82-100 | fl |
| 17 | Average hemoglobin content | 27-34 | pg |
| 18 | Average hemoglobin concentration | 316-354 | g/L |
| 19 | red blood cell distribution width | 11.0-15.5 | % |
| 20 | mean platelet volume | 7.4-12.5 | fl |
| 21 | thrombocytocrit | 0.12-0.35 | % |
| 22 | Average distribution width of platelets | 10.0-30.0 | % |
| **Table S2B.** Twenty-three biochemical analytes | | | |
| Number | Check project | reference range | unit |
| 1 | alanine aminotransferase | 9.0-50.0 | U/L |
| 2 | total protein | 65.0-85.0 | g/L |
| 3 | albumin | 40.0-55.0 | g/L |
| 4 | globulin | 20.0-40.0 | g/L |
| 5 | A/G | 1.2-2.4 |  |
| 6 | total bilirubin | <23.0 | umol/L |
| 7 | direct bilirubin | <8.0 | umol/L |
| 8 | indirect bilirubin | <15.0 | umol/L |
| 9 | AST | 15.0-40.0 | U/L |
| 10 | alkaline phosphatase | 45.0-125.0 | U/L |
| 11 | glutamyl transpeptidase | 10.0-60.0 | U/L |
| 12 | carbon dioxide combining power | 22.0-29.0 | mmo1/L |
| 13 | urea | 3.6-9.5 | mmol/L |
| 14 | creatinine | 57.0-111.0 | umol/L |
| 15 | Uric Acid | 208.0-428.0 | umol/L |
| 16 | GLU | 3.90-6.10 | mmo1/L |
| 17 | creatine kinase | 50.0-310.0 | U/L |
| 18 | Hydroxybutyrate Dehydrogenase | 72.0-182.0 | U/L |
| 19 | lactic dehydrogenase | 120.0-250.0 | U/L |
| 20 | Creatine Kinase Isoenzyme | 0-24.0 | U/L |
| 21 | potassium | 3.5-5.3 | mmol/L |
| 22 | sodium | 137.0-147.0 | mmol/L |
| 23 | chlorine | 99.0-110.0 | mmol/L |
