# Supplementary material for: Clinical characteristics and epidemiological analysis of 23 cases of tick-borne rickettsiosis in Xinjiang Uygur Autonomous Region
Source: Front Cell Infect Microbiol. 2026 Jul 8;16:1865543. doi: 10.3389/fcimb.2026.1865543 (PMC13388217; doi:10.3389/fcimb.2026.1865543)
Supplement: Supplementary Table 3 — Sample pooling strategy for 16S rRNA gene sequencing. [file Table3.docx]

**Supplementary Table S3.** Sample pooling strategy for 16S rRNA gene sequencing.

| Group | Number of mixed blood samples | Pathogen detected by nPCR | Clinical symptom status |
| --- | --- | --- | --- |
| RX1 | 3 | *Rickettsia raoultii* | Symptomatic (mild to moderate) |
| RX2 | 2 | *Rickettsia raoultii* | Symptomatic (mild to moderate) |
| RX3 | 3 | *Rickettsia raoultii* and *Rickettsia sibirica* | Asymptomatic |
| RX4 | 6 | *Rickettsia sibirica* | Symptomatic (mild to moderate) |
| RX5 | 3 | *Rickettsia raoultii* | Symptomatic (mild to moderate) |
| RX6 | 3 | *Rickettsia raoultii* and *Rickettsia sibirica* | Asymptomatic |
| RX7 | 10 | Negative | Symptomatic (mild fever, 37–37.5°C) |

**Note:**A total of 30 patients selected from the 70 tick-bitten cases were pooled into 7 groups for 16S rRNA V3/V4 sequencing. Groups were defined by two criteria: (1) nPCR-confirmed Rickettsia infection status (R. raoultii, R. sibirica, mixed, or negative) and (2) presence or absence of clinical symptoms at the time of blood collection. RX3 and RX6 both consisted of asymptomatic patients infected with Rickettsia spp., but included different sets of individuals. RX7 served as a non-infected symptomatic control group.
