# Supplementary material for: Clinical characteristics and epidemiological analysis of 23 cases of tick-borne rickettsiosis in Xinjiang Uygur Autonomous Region
Source: Front Cell Infect Microbiol. 2026 Jul 8;16:1865543. doi: 10.3389/fcimb.2026.1865543 (PMC13388217; doi:10.3389/fcimb.2026.1865543)
Supplement: Supplementary Table 4 — Serological examination and nPCR results of 70 patients. [file Table4.docx]

**Supplementary Table S4.** Serological examination and nPCR results of 70 patients.

1. Patients with confirmed SFGR infection (nPCR positive, n=23)

| Number | Serology (SFGR IgG) | nPCR | Tick bite to consultation (days) |
| --- | --- | --- | --- |
| 1 | － | + | 6 |
| 2 | － | + | 16 |
| 3 | － | + | 22 |
| 4 | + | + | 5 |
| 5 | － | + | 2 |
| 6 | － | + | 2 |
| 7 | － | + | 7 |
| 8 | － | + | 5 |
| 9 | / | + | 6 |
| 10 | / | + | 13 |
| 11 | + | + | 5 |
| 12 | / | + | 2 |
| 13 | － | + | 25 |
| 14 | － | + | 5 |
| 15 | － | + | 10 |
| 16 | / | + | 7 |
| 17 | / | + | 8 |
| 18 | / | + | 11 |
| 19 | / | + | 20 |
| 20 | － | + | 2 |
| 21 | － | + | 11 |
| 22 | / | + | 7 |
| 23 | / | + | 13 |

1. Patients without SFGR infection (nPCR negative, n=47)

| Number | Serology (SFGR IgG) | nPCR | Tick bite to consultation (days) |
| --- | --- | --- | --- |
| 24 | / | － | 13 |
| 25 | / | － | 7 |
| 26 | / | － | 17 |
| 27 | / | － | 10 |
| 28 | / | － | 7 |
| 29 | / | － | 3 |
| 30 | － | － | 1 |
| 31 | － | － | 4 |
| 32 | － | － | 5 |
| 33 | + | － | 1 |
| 34 | + | － | 1 |
| 35 | － | － | 1 |
| 36 | － | － | 1 |
| 37 | － | － | 1 |
| 38 | － | － | 1 |
| 39 | － | － | 1 |
| 40 | － | － | 1 |
| 41 | － | － | 1 |
| 42 | － | － | 1 |
| 43 | + | － | 4 |
| 44 | － | － | 1 |
| 45 | + | － | 4 |
| 46 | － | － | 1 |
| 47 | + | － | 1 |
| 48 | － | － | 1 |
| 49 | + | － | 4 |
| 50 | － | － | 1 |
| 51 | － | － | 1 |
| 52 | － | － | 14 |
| 53 | + | － | 7 |
| 54 | － | － | 1 |
| 55 | － | － | 1 |
| 56 | － | － | 1 |
| 57 | － | － | 1 |
| 58 | － | － | 4 |
| 59 | － | － | 10 |
| 60 | － | － | 2 |
| 61 | + | － | 4 |
| 62 | － | － | 1 |
| 63 | － | － | 3 |
| 64 | + | － | 1 |
| 65 | － | － | 3 |
| 66 | － | － | 11 |
| 67 | － | － | 3 |
| 68 | + | － | 1 |
| 69 | － | － | 1 |
| 70 | － | － | 4 |

**Note:** “+”: Positive; “－”: Negative; “/”: No result due to serum samples missing.
