# Supplementary material for: Clinical characteristics and epidemiological analysis of 23 cases of tick-borne rickettsiosis in Xinjiang Uygur Autonomous Region
Source: Front Cell Infect Microbiol. 2026 Jul 8;16:1865543. doi: 10.3389/fcimb.2026.1865543 (PMC13388217; doi:10.3389/fcimb.2026.1865543)
Supplement: Supplementary Table 5 — The Laboratory Test Results of 9 hospitalized patients pre- and post-treatment. [file Table5.docx]

**Supplementary Table S5.** The Laboratory Test Results of 9 hospitalized patients pre- and post-treatment.

| Case |  | CRP  (<6.0) | ESR (0-20) | Neutrophils (%) (40-75%) | WBC （3.5-9.5×10^9^） | Lymphocytes (%)  (20-50%) | Platelet  (100-300) | Alb (40-55) | LDH (120-250) | CK  (30-135) | ALT (5-40) | AST  (8-40) | PCT  (<0.05) | Eosinophil  (0.62-0.68) |
| --- | --- | --- | --- | --- | --- | --- | --- | --- | --- | --- | --- | --- | --- | --- |
| 10 | Before | 35.19↑ | 28.00 | 79.8↑ | 10.1↑ | 14.3↓ | 209.00 | 36↓ | 274↑ | 59.00 | 175↑ | 152↑ | 0.14↑ | 0.01↓ |
|  | After | 1.41 | 10.00 | 54.10 | 4.00 | 32.30 | 207.00 | 40.60 | 203.00 | 58.00 | 36.00 | 25.00 | 0.05 | 0.40 |
| 11 | Before | 14.8↑ | 32.00 | 86.2↑ | 32.2↑ | 5.3↓ | 245.00 | 35↓ | 178.00 | 60.00 | 65↑ | 50.8↑ | 0.03 | 0.02↓ |
|  | After | 2.00 | 15.00 | 74.00 | 7.10 | 29.00 | 234.00 | 45.00 | 189.00 | 77.00 | 10.00 | 11.30 | 0.03 | 0.11 |
| 14 | Before | 27.3↑ | 23.1↑ | 89.5↑ | 10.27↑ | 20.00 | 247.00 | 37↓ | 463↑ | 69.00 | 100↑ | 92↑ | 0.04 | 0.00↓ |
|  | After | 0.27 | 17.00 | 63.60 | 8.50 | 23.00 | 198.00 | 44.20 | 187.00 | 77.00 | 12.90 | 21.80 | 0.04 | 0.38 |
| 15 | Before | 1.84 | 2.00 | 55.00 | 10.3↑ | 5.3↓ | 232.00 | 39.7↓ | 178.00 | 70.00 | 15.00 | 25.00 | 0.01 | 0.01 |
|  | After | 7.83 | 13.00 | 50.00 | 3.80 | 28.50 | 245.00 | 41.40 | 156.00 | 58.00 | 18.00 | 27.00 | 0.01 | 0.20 |
| 17 | Before | 92.1↑ | 13.30 | 89.7↑ | 20.1↑ | 15.3↓ | 200.00 | 34↓ | 281↑ | 310↑ | 56.2↑ | 183↑ | 0.9↑ | 0.01↓ |
|  | After | 6.20 | 13.00 | 57.00 | 4.00 | 45.00 | 208.00 | 40.30 | 157.00 | 79.00 | 34.00 | 17.00 | 0.05 | 0.07 |
| 18 | Before | 100.00↑ | 27↑ | 94.10 ↑ | 14.42↑ | 10.3↓ | 96↓ | 26↓ | 263↑ | 215↑ | 45↑ | 50↑ | 0.85↑ | 0.01↓ |
|  | After | 5.20 | 11.50 | 55.60 | 7.74 | 33.00 | 266.00 | 33.93 | 200 | 70.00 | 37.00 | 40.00 | 0.05 | 0.04 |
| 19 | Before | 14.6↑ | 8.50 | 87.7↑ | 10.3↑ | 13.8↓ | 267.00 | 31.4↓ | 341↑ | 206↑ | 145↑ | 52↑ | 0.01 | 0.02↓ |
|  | After | 3.80 | 8.60 | 61.90 | 9.30 | 32.20 | 257.00 | 47.00 | 139.00 | 63.70 | 10.30 | 22.1 | 0.02 | 0.20 |
| 20 | Before | 96↑ | 11.70 | 85.4↑ | 7.10 | 7.3↓ | 267.00 | 24.2↓ | 891↑ | 103.00 | 181↑ | 382↑ | 6.72↑ | 0.15↓ |
|  | After | 4.00 | 11.50 | 58.40 | 6.90 | 31.70 | 234.00 | 45.00 | 163.00 | 55.00 | 8.60 | 13.60 | 0.04 | 0.38 |
| 22 | Before | 10.45↑ | 9.90 | 88↑ | 5.00 | 6.7↓ | 276.00 | 27.7↓ | 381↑ | 206↑ | 98↑ | 103↑ | 0.03 | 0.01↓ |
|  | After | 2.20 | 10.60 | 50.40 | 5.12 | 27.40 | 265.00 | 45.50 | 204.00 | 115.00 | 23.00 | 8.90 | 0.02 | 0.50 |
